# Trial Designs Beyond Traditional Randomized Controlled Trials: Applications and Appropriateness in Osteoarthritis Research

**DOI:** 10.7759/cureus.91163

**Published:** 2025-08-28

**Authors:** Kalpana Singh, Liyan Dsouza, Treesa Thomas, Abdulqadir J Nashwan

**Affiliations:** 1 Biostatistics Unit, Nursing and Midwifery Research Department, Hamad Medical Corporation, Doha, QAT; 2 College of Medicine, Hamad Medical Corporation, Doha, QAT; 3 Nursing and Midwifery Research Department, Hamad Medical Corporation, Doha, QAT

**Keywords:** clinical trial design, osteoarthritis, patient-centered research, randomized controlled trials, real-world evidence

## Abstract

The prevalence of osteoarthritis (OA) has been rising, and despite the many years of scientific research and evidence collection, OA remains a medical issue where effective interventions are limited due to the complexity and heterogeneity of the disease, the disconnect between symptoms and structural changes, and potential demands from an authoritative standpoint. Though randomized controlled trials (RCTs) are considered the gold standard in research, they might not be as applicable in the context of OA research. Hence, this editorial aims to investigate alternative trial designs and highlight the strengths and limitations of their utilization to develop effective interventions and treatments, which may ultimately lower the OA burden and improve patient outcomes.

## Editorial

Introduction

Globally, around 528 million people were noted to be living with osteoarthritis (OA) in 2019, and its prevalence is assumed to rise due to the increasing rates of injury and obesity, and with higher ageing populations [1]. Despite decades of research progressing our understanding of the molecular basis of OA, and the persisting unmet medical needs of OA patients, effective treatments that address the structure and symptoms of OA are yet to be brought to the market. Several challenges exist that hinder progress in this area, including the slow progression of the disease, the perceived disconnect between symptoms and structural changes [2], and the possible requirements for health authority-accepted endpoints [2]. Randomized controlled trials (RCTs), while considered the gold standard in research, are not always feasible or appropriate, especially for complex diseases like OA, which is characterized by diverse patient populations, varying symptoms, and unpredictable disease progression [3,4].

Thus, there is an increasing need for more efficient and flexible trial designs that are better suited to answer complex real-world settings in the context of OA research. A few examples of these alternative designs include pragmatic trials, adaptive trials, cohort studies, registry-based trials, and cluster randomized trials.

Pragmatic Trials

Pragmatic clinical trials (PCTs) are designed to investigate the full spectrum of interventions in the context of a routine clinical setting to optimize generalizability and applicability. The outcomes measured are wide, though largely patient-centered [5]. The measure of external validity is a priority in PCTs to study interventions for their application in broad-based and clinical practice situations [6]. An assessment of 34 manual therapy knee OA trial designs for their explanatory-pragmatic fit conducted by Adams et al. [7] concluded that to analyze the effectiveness of manual therapy for the treatment of knee OA, higher pragmatic trial designs might improve results implementation and application from their research studies. By increasing the diversity of the study population, incorporating more clinics, more pragmatic intervention protocols, and comparing interventions and treatments that appropriately match routine clinical care, OA will be able to improve and increase the generalizability of study conclusions for manual therapy trials [7].

As PTs aim to examine several interventions in varying settings and compare treatment effectiveness, they often require larger sample sizes and longer follow-ups to draw reliable and reusable conclusions, hence making PTs more expensive [5]. Though external validity is strengthened in PTs, internal validity is often compromised in the process [8]. Absence of a control or placebo study group might lead to a higher risk of participant withdrawal, thus affecting the participant-reported outcome measure. Uncertainty in results and potential increase of risk in type II errors due to differences in treatment responses in heterogeneous patient groups and differing adherence levels could cause an oversight in identifying the actual treatment effect. Furthermore, these limitations could make researchers disinclined to consider PTs as the risk of negative findings is high, further limiting the application and awareness of these trial designs [8]. For scientific diligence, trial inclusion and exclusion criteria, protocol adherence by practitioners, appropriate comparison groups, and follow-up are all necessary to measure outcome changes. Thus, challenges arise in the criteria involving increasing generalizability and reducing bias, and resolution depends on the parameters of individual studies and the research question itself [9].

Adaptive Trials

Adaptive trial designs (ADs) facilitate preplanned modifications to trial procedures or statistical methods subsequent to initiation, provided such alterations do not jeopardize the study's validity or integrity. This allows for increased flexibility and efficiency, with reduced duration of study [10], reduced use of resources, and limits participant allocation to an inadequate intervention [11]. However, any planned adaptations must be specified in advance and based on accumulating study data [10].

A defining trait of adaptive designs is that modifications made to the ongoing trial are all based on interim data analyses with crucial preservation of the validity and integrity of the study [12]. Areas of modification in adaptive trials include complete sample size, allocation ratio, and eligibility criteria, as well as adding or dropping certain treatment arms, or extending a trial from phase II to phase III [11]. Adaptive trial designs are widely used in drug evaluation studies, though their applications are beginning to spread. Outside of OA, ADs have been used extensively with significant results from research studies on topics such as severe chronic angina, telmisartan and insulin resistance in HIV (TAILoR), and adverse karyotype acute myeloid leukemia (AML) [12-15].

Although ADs could reduce the study duration, resource use, the number of participants allocated to inferior treatment arms, and generally improve the chances of trial success, their potential to generate inefficiencies due to poor planning may hinder their application in research. Hence, any adaptation or modification to the study must be rigorously assessed in terms of risk-benefit. The potential ethical and scientific gains of the study should exceed the risk of inefficiencies and bias generated in the trial [11]. Due to the requirement of such rigorous processes in ADs, they are considered more complicated and challenging to plan and execute. Interpretation of results is more complex; the risk of confounding through the introduction of a trial modification, and investigator-driven bias are other limitations potentially arising in the design and execution of adaptive trials [16].

Cohort Studies

Cohort studies entail the selection of participants according to their exposure status and involve longitudinal follow-up to determine the occurrence of the outcome of interest [17]. They are especially useful in clinical research when previous evidence indicates a link between exposure and outcome, and when the time interval between exposure and disease development is reasonable [18]. These trial designs allow for the observation of natural history and disease progression, and enable the calculation of incidence rate and cumulative incidence, hazard ratio, and relative risk. Cohort studies cannot definitively establish causality; however, they do provide information on the strength of association between the outcome and possible risk factors [18]. Their application in OA research includes the Worldwide Collaboration on OsteoArthritis prediCtion for the Hip (World COACH) initiative that uses ‘harmonized’ collective data on imaging, clinical findings, genetics, biomarkers, and lifestyle to offer unique possibilities for the development of individual or personalized risk prediction models for hip OA and could potentially optimize imaging analysis methods of the hip [19].

Despite their strengths, cohort studies have several limitations. They often require large sample sizes, especially for rare exposures, making them expensive and time-consuming [20]. Loss to follow-up can lead to attrition bias, particularly if withdrawing participants have different baseline risks than those who stay, which can limit the generalizability of the results. Moreover, information bias could be introduced in cases where any difference in the quality of exposure measurement between the two groups, exposed and non-exposed, and hence, might lead to a skewing of results [20]. Non-randomization of participants into specific subgroups during a comparison of the interest groups of a particular outcome could lead to several confounding variables complicating the data analysis and interpretation process [21]. In such cases, it is suggested to use ‘Cox proportional hazards regression’ that generates a hazard ratio that can adjust for confounding as well as consider the differing follow-up duration and the occurrence time of the outcome of interest [21].

Registry-Based Trials

Registry-based trials leverage existing patient registries to answer specific research questions, providing a practical method for clinical research [22]. Their applications involve evaluating alternative interventions currently used in clinical settings, comparing their effectiveness, investigating indications and new signals of approved interventions, and analyzing non-pharmacological interventions in research [23]. For instance, Jonsson et al. investigated whether a first-line three-month intervention for knee or hip osteoarthritis (OA) pain, delivered either face-to-face or digitally, produced meaningful differences [24]. Their Swedish registry-based cohort study showed clinically relevant improvements in both groups, with a modest additional benefit in the digital group. Importantly, the results supported the use of both methods, thereby increasing treatment access for patients with knee or hip OA [24].

Registry-based trials are advantageous due to their efficient recruitment and follow-up processes, shorter study durations, and utilization of existing infrastructure and networks. These qualities contribute to cost-effectiveness, facilitate quick participant identification, streamline data collection, and support long-term follow-up [23,25]. Registry-based trials are cost-effective due to the existing personnel, infrastructure, and network, and have high generalizability due to the inclusion of a wider participant range. Hence, study outcomes would be reflective of a real-world setting [23]. A limitation of this trial design includes the precision of a registry in answering the research question posed. Others include limited registry size, data availability, possible selection bias, and registry adaptability or flexibility. Data quality in a registry can impact the reliability, internal validity, and completeness of the study. Furthermore, reduced accessibility to clinical registries due to limited ethical and infrastructural conditions, such as data security, is a common limitation to these trials [23].

Cluster Randomized Trial

With cluster randomized trials (CRTs), pre-existing groups of individuals are allocated to treatment arms randomly. They are particularly useful when interventions are delivered at the cluster level or when individual randomization is not feasible [26]. CRTs may effectively examine guideline recommendations, new care standards, and other hospital-wide, practice-wide, or system-wide changes that may impact patient outcomes [27]. These designs can allow for higher acceptability and logistical convenience when presented to a whole population rather than individuals [28]. For example, an ongoing research study conducted by Mills et al. uses a mixed-method cluster-randomized implementation trial to evaluate the impact of ‘educational reminder messages’ displayed under patient X-ray reports, regardless of whether patient-facing infographic information was provided [29]. This study is aimed toward patients with early knee OA who present to accredited exercise professionals to potentially devise a strategy to increase the number of early knee OA patients to take on benchmark evidence-based practice in healthcare [29].

Limitations to CRTs include contamination from participant contact with individuals outside the cluster, which could impact in-study responses and cause issues in the CRT [28,30]. Hayes and Moulton suggest a well-separated cluster selection and, in cases of larger geographical clusters, creating ‘buffer zones’ so that the two groups do not have a common boundary, hence reducing the degree of contamination in a CRT [28]. Furthermore, insufficient sampling in a CRT could lead to higher random error and lower the study’s power, hence reducing the accuracy of measuring the effect. Inter-cluster correlations can occur through individuals’ interactions, variations in response to intervention, and clustering of similar population characteristics, and the extent of this correlation depends on the size, nature, and the presence of other clusters, as discussed by Hayes and Moulton [28]. Other limitations to consider include cost efficiency, statistical complexity, reduced power of CRT compared to RCTs, selection bias, generalizability, and potential treatment arm imbalance [28,30,31].

Conclusion

The key message of this editorial to the research community is to highlight the importance of selecting the most appropriate trial design based on the specific research question and the complexities of OA that is being investigated. Researchers are encouraged to consider these alternative designs to advance OA research more efficiently, ultimately leading to the development of more effective, personalized treatments for patients. Additionally, interdisciplinary collaboration among partnerships and scientists with health authorities, patients, and payers may potentially aid in overcoming challenges and lead to advancements in the field.

This editorial recognizes the complexity of OA and that no single research trial design can effectively address all aspects of the disease's multifaceted nature. However, the practical applications of each trial design mentioned illustrate their relevance in a real-world context. While RCTs will continue to play a central role in clinical research, alternative trial designs may be considered as essential tools for addressing the complexity of OA. These designs can offer enhanced flexibility and provide real-world relevancy and efficiency in specific disease contexts, hence contributing to the potential development of more tailored and effective treatments for patients with OA. Therefore, understanding the strengths and limitations of each trial design is crucial for advancing the field of OA research to enhance disease treatment and management and patient care.

## References

[1] (2025). WHO. Osteoarthritis. https://www.who.int/news-room/fact-sheets/detail/osteoarthritis.

[2] Felson DT, Neogi T (2018). Emerging treatment models in rheumatology: challenges for osteoarthritis trials. Arthritis Rheumatol.

[3] Saldanha IJ, Adam GP, Bañez LL (2022). Inclusion of nonrandomized studies of interventions in systematic reviews of interventions: updated guidance from the Agency for Health Care Research and Quality Effective Health Care program. J Clin Epidemiol.

[4] Towheed TE (2005). Systematic review of therapies for osteoarthritis of the hand. Osteoarthritis Cartilage.

[5] Patsopoulos NA (2011). A pragmatic view on pragmatic trials. Dialogues Clin Neurosci.

[6] Delitto A (2016). Pragmatic clinical trials: implementation opportunity, or just another fad?. Phys Ther.

[7] Adams KR, Famuyide AO, Young JL, Maddox CD, Rhon DI (2024). Pragmatism in manual therapy trials for knee osteoarthritis: a systematic review. Arch Physiother.

[8] Lyngbakken MN, Paulsen A, Sethupathy A, Hesselberg Ø, Grimsgaard S, Hagen K (2021). Pragmatic trials - what are they? [Article in Norwegian]. Tidsskriftet.

[9] Ali SA, Kloseck M, Lee K, Walsh KE, MacDermid JC, Fitzsimmons D (2018). Evaluating the design and reporting of pragmatic trials in osteoarthritis research. Rheumatology (Oxford).

[10] Mahajan R, Gupta K (2010). Adaptive design clinical trials: methodology, challenges and prospect. Indian J Pharmacol.

[11] Thorlund K, Haggstrom J, Park JJ, Mills EJ (2018). Key design considerations for adaptive clinical trials: a primer for clinicians. BMJ.

[12] Pallmann P, Bedding AW, Choodari-Oskooei B (2018). Adaptive designs in clinical trials: why use them, and how to run and report them. BMC Med.

[13] Chaitman BR, Pepine CJ, Parker JO (2004). Effects of ranolazine with atenolol, amlodipine, or diltiazem on exercise tolerance and angina frequency in patients with severe chronic angina: a randomized controlled trial. JAMA.

[14] Pushpakom SP, Taylor C, Kolamunnage-Dona R (2015). Telmisartan and Insulin Resistance in HIV (TAILoR): protocol for a dose-ranging phase II randomised open-labelled trial of telmisartan as a strategy for the reduction of insulin resistance in HIV-positive individuals on combination antiretroviral therapy. BMJ Open.

[15] Giles FJ, Kantarjian HM, Cortes JE (2003). Adaptive randomized study of idarubicin and cytarabine versus troxacitabine and cytarabine versus troxacitabine and idarubicin in untreated patients 50 years or older with adverse karyotype acute myeloid leukemia. J Clin Oncol.

[16] Park JJ, Thorlund K, Mills EJ (2018). Critical concepts in adaptive clinical trials. Clin Epidemiol.

[17] Setia MS (2016). Methodology series module 1: cohort studies. Indian J Dermatol.

[18] Wang X, Kattan MW (2020). Cohort studies. Design, analysis, and reporting. Chest.

[19] van Buuren MM, Riedstra NS, van den Berg MA (2024). Cohort profile: Worldwide Collaboration on OsteoArthritis prediCtion for the Hip (World COACH) - an international consortium of prospective cohort studies with individual participant data on hip osteoarthritis. BMJ Open.

[20] Alexander LK, Lopes B, Ricchetti-Masterson K, Yeatts KB (2014). Cohort studies. The Eric Notebook.

[21] Andrade C (2022). Research design: cohort studies. Indian J Psychol Med.

[22] (2021). European Medicines Agency (EMA). Guideline on registry-based studies. https://www.ema.europa.eu/en/guideline-registry-based-studies-scientific-guideline.

[23] Zazryn T, Clavisi O, Skiba M (2024). A Guide for Registry-Based Trials. https://www.health.gov.au/sites/default/files/2025-05/a-guide-for-registry-based-trials-national-clinical-quality-registry-program.pdf.

[24] Jönsson T, Dell'Isola A, Lohmander LS, Wagner P, Cronström A (2022). Comparison of face-to-face vs digital delivery of an osteoarthritis treatment program for hip or knee osteoarthritis. JAMA Netw Open.

[25] Li G, Sajobi TT, Menon BK (2016). Registry-based randomized controlled trials- what are the advantages, challenges, and areas for future research?. J Clin Epidemiol.

[26] (n.d). Queen Mary University of London. Cluster randomised trials. Pragmatic Clinical Trials Unit. https://www.qmul.ac.uk/pctu/studies/methods-research/cluster-randomised-trials/.

[27] Heagerty PJ (2023). Cluster randomized trials. Experimental Designs and Randomization Schemes. Rethink Clin Trials Living Textb Pragmatic Clin Trials. Published online March 27.

[28] Hayes RJ, Moulton LH (2009). Cluster randomised trials. Quantifying impact. LSHTM. Centre for Evaluation. January.

[29] Mills K, Bowden JL, Boland R, Pardey M, Descallar J, Naylor JM (2023). Taking the first step: protocol for a cluster randomised implementation trial comparing strategies on access to exercise programmes for people with knee osteoarthritis. BMJ Open.

[30] World Health Organization (2022). WHO Guidance on Research Methods for Health Emergency and Disaster Risk Management, Revised 2022.

[31] Malliou N, Antoniou P, Antonopoulou K (2023). Pos0192-pare towards a new approach on research designs: patients and healthcare professionals co-creating and co-developing a mobile application for the self-management of chronic pain in osteoarthritis. The Tous Include Project. Ann Rheum Dis.

